# Interferon-γ Induced by *In Vitro* Re-Stimulation of CD4+ T-Cells Correlates with *In Vivo* FMD Vaccine Induced Protection of Cattle against Disease and Persistent Infection

**DOI:** 10.1371/journal.pone.0044365

**Published:** 2012-09-28

**Authors:** Yooni Oh, Lucy Fleming, Bob Statham, Pip Hamblin, Paul Barnett, David J. Paton, Jong-Hyeon Park, Yi Seok Joo, Satya Parida

**Affiliations:** 1 Pirbright Laboratory, Institute for Animal Health, Surrey, United Kingdom; 2 National Veterinary Research and Quarantine Service, Anyang, Republic of Korea; Centers for Disease Control and Prevention, United States of America

## Abstract

The immune defense against FMDV has been correlated to the antibody mediated component. However, there are occasions when some animals with high virus neutralising (VN) antibody are not protected following challenge and some with low neutralising antibody which do not succumb to disease. The importance of cell mediated immunity in clinical protection is less clear and so we investigated the source and production of interferon-gamma (IFN-γ) in re-stimulated whole blood of FMDV immunized cattle and its correlation to vaccine induced protection and FMDV persistence. We were able to show a positive correlation between IFN-γ response and vaccine induced protection as well as reduction of long term persistence of FMD virus. When combining this IFN-γ response in re-stimulated blood with virus neutralizing antibody titer in serum on the day of challenge, a better correlation of vaccine-induced protection with IFN-γ and VN antibody was predicted. Our investigations also showed that CD4+ T-cells are the major proliferating phenotype and IFN-γ producing cells.

## Introduction

Foot-and-mouth disease (FMD) is an economically devastating and highly contagious disease of domestic and wild cloven-hoofed animals including cattle, sheep, goats and pigs. The causative agent is *Foot-and-mouth disease virus* (FMDV) which is a single-stranded positive-sense RNA virus belonging to the genus *Aphthovirus* in the family *Picornaviridae*. Although FMD does not result in high mortality in adult animals, the disease has debilitating effects including weight loss, decrease in milk production and loss of draught power. The virus can continue to be detected in the oropharynx of up to 50% of cattle beyond 28 days after initial infection and such animals are termed FMDV carriers [1]. FMD can be controlled by vaccination using chemically inactivated and adjuvanted cell culture grown virions that can reduce virus replication leading to prevention of disease and blocking of transmission [2].

The immune defence against FMD has been correlated to the antibody mediated component and humoral antibody responses have been considered to be the most important factor in conferring protection against FMD [3]. Measuring the efficacy of FMD vaccines relies on either direct potency tests by challenge of vaccinated animals (usually cattle) or indirect assessment based on the serological response induced by vaccination. However, despite a good correlation between serum antibody titres and protection, there are instances where animals with medium or high neutralising antibody titres are not protected upon challenge with virus [4]. Similarly, animals with low or no detectable virus neutralising antibody on occasion do not succumb to disease [5]. In FMD infection as well as FMD vaccination, B lymphocytes are responsible for humoral antibody production, but a role for T cells in the induction of antibody responses in ruminants has also been suggested, based on the demonstration of FMDV-specific CD4 T-cell proliferative responses following infection or vaccination with virus or a viral peptide [6], [7], [8]. Borca et al. [9] in a murine experimental model and Juleff et al., [10] in a bovine experimental model reported that the protective immune response against FMDV infection is T cell independent. However, there is limited knowledge of the role of cell mediated responses against vaccine induced protection. Interferon-gamma (IFN-γ) has antiviral activity against FMDV [11], [12], [13], [14] and also promotes natural killer (NK) cell and macrophage activation that are likely to contribute to control of FMDV replication and spread within the host [13]. Recently, we have developed an *ex-vivo* IFN-γ assay to measure the quantity of IFN-γ in whole blood of FMDV vaccinated and infected cattle after re-stimulation with inactivated vaccine antigen [15]. Using this IFN-γ assay and virus neutralisation (VN) test, we report on a positive correlation between IFN-γ production and VN titres with vaccine–induced protection in vaccinated cattle on the day of challenge that has potential to help to predict the outcome of a subsequent challenge, in terms of clinical protection and the long term detection of virus (persistent infection) from the oropharynx. Further, we elucidate *in-vitro* that CD4+ T-cells are the major proliferating phenotype and are mainly responsible for IFN-γ production in re-stimulated blood of FMDV vaccinated animals.

## Results

### 1.1 Clinical and virological results

As expected, upon challenge with homologous virus, all the unvaccinated control animals in both the experiments were infected and developed lesions on all four feet and mouth. All vaccinates in the A Malaysia 97 potency test were clinically protected and the vaccine passed with an estimated PD_50_ value ≥32, whereas three vaccinates from the 1/4 dose group and one from the 1/16 dose group of the SAT2 Eritrea potency experiment showed clinical lesions. Despite this, the vaccine passed with an estimated PD_50_ value of 10.

Live virus as well as viral RNA was recovered from the oro-pharyngeal (OP) samples of all the four non-vaccinated control animals of both the experiments. Out of these four, the two SAT2 Eritrea infected animals became carriers whereas virus could not be recovered after 28 days post challenge (dpc) from the two A Malaysia 97 infected animals. Although all vaccinates were clinically protected in the A Malaysia 97 experiment, live virus was isolated at or beyond 12 dpc from two animals in the full dose vaccine group, from three animals in the 1/4 dose group and from all five animals in the 1/16 dose group (Table 1). In contrast, live virus was isolated from all 15 vaccinates in the SAT2 Eritrea experiment on or after 12 dpc (Table 1). Of 10 sub-clinically infected animals detected in the A Malaysia 97 experiment by virus isolation and PCR, one animal, from the full dose and 1/4 dose groups, and three animals from the 1/16 dose group became carriers (Table 1). Similarly, out of the 15 SAT2 Eritrea vaccinates, two from the full dose, two from the 1/4 dose group and four from the 1/16 dose group were scored as carriers (Table 1).

**Table 1:** Summary results of clinical status of A Malaysia 97 and SAT2 experimental animals.

	A MAY 97 Animals	P/NP	C/NC	Virus ≥12dpc	SAT2 Animals	P/NP	C/NC	Virus ≥12dpc
**Full dose**	1	P	C	+	1	P	NC	+
	2	P	NC	+	2	P	NC	+
	3	P	NC	−	3	P	C	+
	4	P	NC	−	4	P	C	+
	5	P	NC	−	5	P	NC	+
**1/4^th^ dose**	6	P	NC	−	6	P	NC	+
	7	P	NC	+	7	P	NC	+
	8	P	C	+	8	NP	C	+
	9	P	NC	−	9	NP	NC	+
	10	P	NC	+	10	NP	C	+
**1/16^th^ dose**	11	P	C	+	11	P	C	+
	12	P	NC	+	12	NP	C	+
	13	P	C	+	13	P	C	+
	14	P	C	+	14	P	NC	+
	15	P	NC	+	15	P	C	+
**Control**	16	NP	NC	+	16	NP	C	+
	17	NP	NC	+	17	NP	C	+

Feet and mouth were checked daily for lesions up to two weeks after challenge to classify animals as clinically protected (P) or non-protected (NP). Detection of virus by either virus isolation or real time RT-PCR in probang samples collected from 28–34 days post challenge (dpc) gave an indication of oro-pharyngeal viral replication/persistence and determined the FMDV carrier status (C, carrier and NC, non-carrier).

### 1.2 Correlation of clinical protection with IFN-γ and virus neutralising (VN) antibody responses on the day of challenge

**Figure 1:**
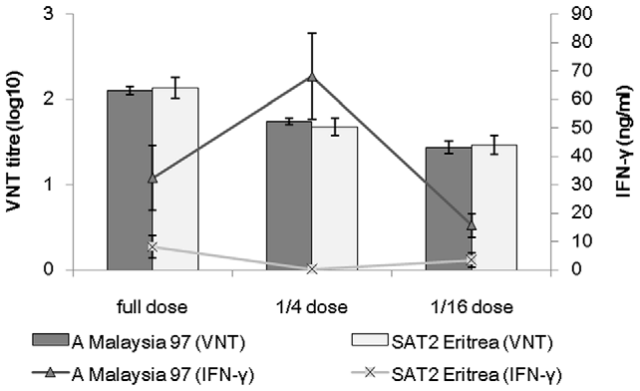
Comparison of virus neutralising antibody titre and IFN-γ response. Comparison of mean group virus neutralising antibody titre (bars) in the serum and mean group IFN-γ response (line) in the re-stimulated (with vaccine antigen) blood of A Malaysia 97 and SAT2 Eritrea cattle on the day of challenge. Error bars represent standard error of the mean.

Within seven days of vaccination, IFN-γ production was observed after re-stimulation of blood in culture with vaccine antigen in all three vaccination groups in the A Malaysia 97 experiment. In contrast, only in the full dose vaccinated animals of the SAT2 Eritrea experiment was an IFN-γ response apparent on the 2^nd^ week after vaccination, whereas the other two groups only produced an IFN-γ response on or after the day of challenge. Mean IFN-γ responses of each individual in the A Malaysia 97 vaccinated group were significantly higher (P = 0.001) than the respective SAT2 Eritrea vaccinated groups (Fig. 1), or after 21 days post vaccination (dpv).

Throughout the post-challenge period, in both experiments, the IFN-γ level was significantly higher (P = 0.001) in the vaccinated animals compared to the control group. A high IFN-γ response was detected after re-stimulation with poke weed mitogen (PWM) as a positive control in all the animals whereas stimulation with PPDA (*M. Avium* PPD) and Baby Hamster Kidney (BHK) cell lysate as a negative control and non-stimulated blood, as a baseline control, induced no IFN-γ response throughout the experiments (data not shown).

**Figure 2:**
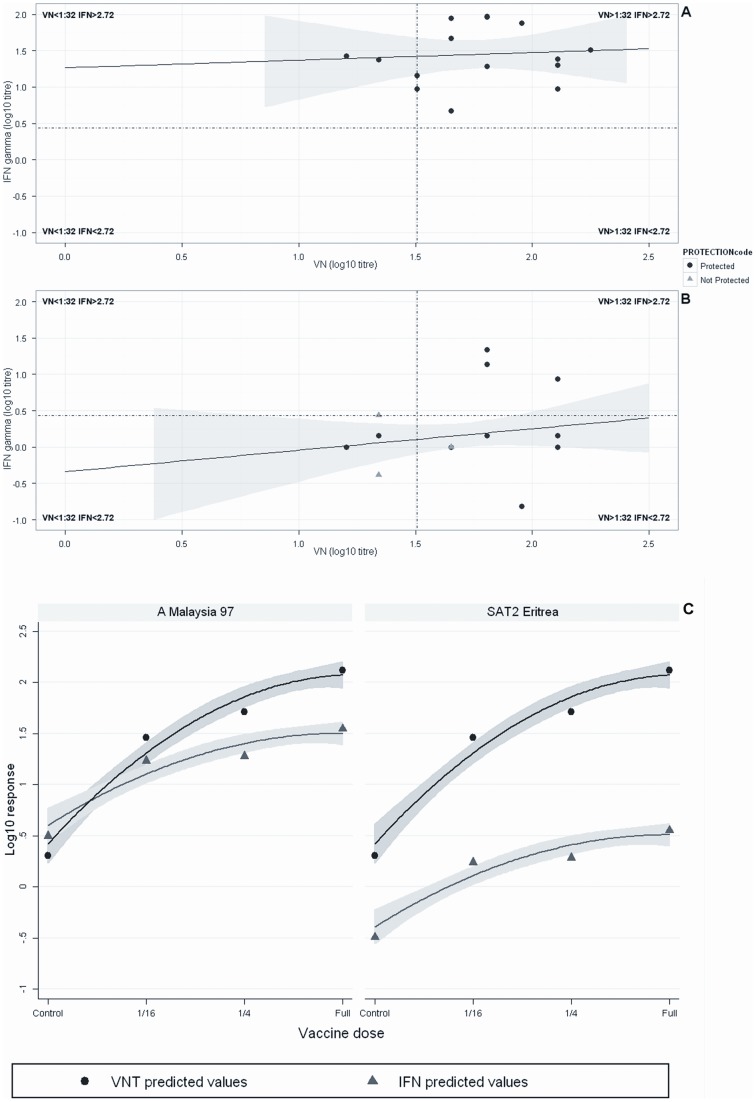
(A & B) Regression analysis between IFN-γ vs VNT responses. Regression analysis between IFN-γ vs VNT responses among 30 vaccinated cattle in A Malaysia 97 (A) and SAT2 Eritrea (B) experiments on the day of challenge. The levels of IFN-γ (ng/ml) did not correlate to the levels of VNT titre in both experiments, where 0.93% and 11.19% of the variability between the IFN-γ and VNT responses were expressed by the model for the A Malaysia 97 and SAT2 Eritrea experiments, respectively. The correlations are expressed by the equations: IFN-γ (log_10_ ng/ml)  = 1.211+0.126 VNT (log_10_ titre), for A Malaysia 97; IFN-γ (log_10_ ng/ml)  = −0.672+0.536 VNT (log_10_ titre), for SAT2 Eritrea. The dotted vertical horizontal lines are set at a low IFN-γ and low VNT threshold for 2.72 (0.434 log_10_) and 1∶32 (1.505 log_10_), respectively. Grey areas indicate 95% Confidence Interval range. (C) Predicted values of IFN-γ vs VNT responses by vaccine dose. Predicted and fitted values for the regression analysis of IFN-γ and VNT responses among 30 cattle vaccinated with different doses of oil-adjuvant inactivated FMDV A Malaysia 97 and SAT2 Eritrea vaccines on the day of challenge. Grey areas indicate 95% Confidence Interval ranges.

On the day of challenge, mean VN titres and IFN-γ responses of the different vaccine dose groups of the A Malaysia 97 experiment were compared (Fig. 1) with the corresponding groups of the SAT2 Eritrea experiment. No significant differences in mean VN titres were observed (P = 0.591, 0.288 and 0.578 for full, 1/4, and 1/16 dose group respectively) between the corresponding groups of animals of both the experiments. In contrast to the VN titres, a significant difference in the IFN-γ response was observed between the full dose (P = 0.019), 1/4 dose (P = 0.000) and 1/16 dose groups (P = 0.008) of the A Malaysia 97 and SAT2 Eritrea cattle (Fig. 1). A base level IFN-γ response was observed in control animals in both the experiments as expected. It is clear from these two animal experiments that IFN-γ response is not dose dependent in contrast to VNT as 1/4^th^ vaccine dose group in A Malaysia 97 experiment produced more IFN-γ response than the full dose group (Fig. 1). Also IFN-γ response is highly variable among the animals in the same vaccine dose group. Further, on the day of challenge a regression analysis was carried out between IFN-γ vs VNT responses among the 30 vaccinated cattle used in the A Malaysia 97 and SAT2 Eritrea experiments to analyse the concordance between these two immunological parameters in predicting protection. The levels of IFN-γ (ng/ml) did not correlate to the levels of VNT titre in both experiments, where 0.93% and 11.19% of the variability between the IFN-γ and VNT responses were expressed by the model for the A Malaysia 97 and SAT2 Eritrea experiments, respectively. The correlations are expressed by the equations: IFN-γ (log_10_ ng/ml)  = 1.211+0.126 VNT (log_10_ titre), for A Malaysia 97; IFN-γ (log_10_ ng/ml)  = −0.672+0.536 VNT (log_10_ titre), for SAT2 Eritrea (Fig. 2a & 2b). A dotted horizontal and a vertical line were set at low IFN-γ (2.72 ng/ml) and low VNT (1∶32) thresholds, respectively. The IFN-γ response and VN titre were considered high when they reached more than 2.72 ng/ml and 1∶32, respectively. These two dotted horizontal and vertical lines divided 15 vaccinated animals in each animal experiment into 4 different groups (Fig. 2a & 2b). In the A Malaysia 97 experiment the majority of protected animals (n = 11) in the upper right hand panel had 39.41 ng/ml mean IFN-γ responses and 1∶89 mean VNT titres. Another four protected animals in the A Malaysia 97 experiment in the upper left hand panel had 37 ng/ml mean IFN-γ responses and 1∶26 mean VN titres. In the SAT2 Eritrea experiment, 4 protected animals in the upper right hand panel had 13.44 ng/ml mean IFN-γ responses and 1∶153 mean VNT titres. Four animals in in the SAT2 Eritrea experiment in the lower left hand panel were found with 1.14 ng/ml mean IFN-γ responses and 1∶21 mean VN titre responses, though two of them were protected. The remaining 7 animals placed in the lower right hand panel revealed 0.44 ng/ml mean IFN-γ responses and 1∶78 mean VN titre. Two animals from this group produced 0.01 ng/ml of IFN-γ and 1∶45 VN titre and were not protected. The 4 clinically affected animals had a mean IFN-γ response of 0.79 ng/ml and mean VN titre of 1∶34.

Furthermore, a mixed effect linear model was computed for predicting the IFN-γ and VNT responses of different vaccine dose groups in both the A Malaysia 97 and the SAT2 Eritrea experiments. The dose and experiment variables were treated as nested structure and added as random effect into the model. As the results indicate, the behaviour of the tests differs according to the type of vaccine used. Although the VNT response in either the A Malaysia 97 and SAT2 Eritrea is comparable and described by a significant increase according to the vaccine dose used (1.456, 1.713 and 2.120 for 1/16, 1/4 and full vaccine doses, respectively), the analysis indicates a lower IFN-γ response in the SAT2 Eritrea experiment (0.237, 0.283 and 0.550 for 1/16, 1/4 and full vaccine doses, respectively) compared to the A Malaysia 97 (1.231, 1.276, 1.543 for the 1/16, 1/14 and full vaccine doses, respectively) and lower in comparison with the VNT (Fig. 2c). Furthermore, the IFN-γ responses for the 1/4 and 1/16 vaccine dose groups in each vaccine experiment were almost identical and not significantly different from the full dose group (p = 0.368).

**Figure 3:**
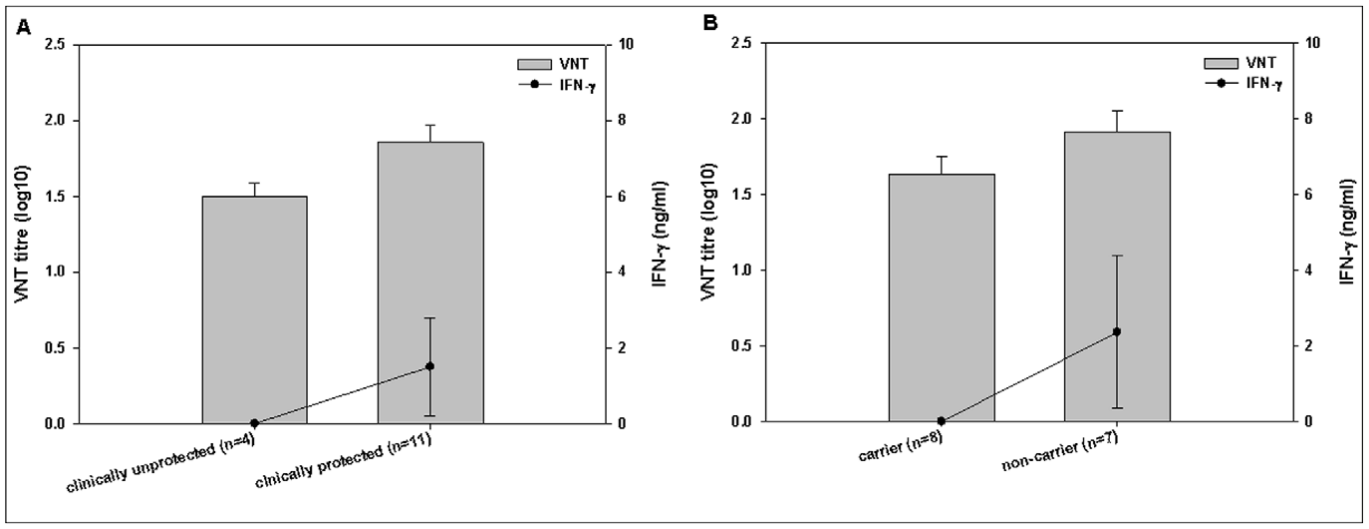
Comparison of mean IFN-γ response and mean VNT responses. Comparison of mean IFN-γ response (line) and mean VNT responses (bar) between clinically protected and non-protected animals (A) and carrier and non-carrier animals (B) on the day of challenge. Error bars represent the standard error of the mean. Data from SAT2 Eritrea experiments were used to develop A and B.

To establish a correlation between clinical protection, IFN-γ and VN antibody responses on the day of challenge, all 15 vaccinated animals from the SAT2 Eritrea experiment were re-grouped into clinically unprotected, clinically protected, carrier and non-carrier, and their mean group values of IFN-γ response and VN antibody titre were also compared. A significant difference in the level of both IFN-γ (P = 0.001) and VN antibody (P = 0.029) was observed between clinically unprotected and clinically protected groups (Fig. 3A).

Out of a total of 4 clinically unprotected animals, three became carriers whereas one did not. Out of 11 clinically protected animals, 5 became carriers whereas 6 were non-carriers. Therefore, a total of 8 carriers and 7 non carriers were confirmed in the SAT2 Eritrea vaccinated animal. The IFN-γ response was found to be significantly different (P = 0.001) when compared between the total carrier (n = 8) and non-carrier (n = 7) animals whereas for the VN antibody titre there was no significant difference (P = 0.156) between these two groups, although a high mean VNT was observed for the non-carrier group (Fig. 3B). All the 15 vaccinated cattle in the A Malaysia 97 experiment had a mean VNT titre of 1∶72 and a mean IFN-γ response of 38.77 and were clinically protected. As no clinically infected animals were available in this experiment, a correlation protection between VNT and IFN-γ response with infection was not established.

### 1.3 Validation of whole blood re-stimulated assay with standard immunological assays (PBMC re-stimulation, ELISpot and lympho proliferation assay)

#### 1.3.1 Comparison of IFN-γ production in re-stimulated PBMC and whole blood assays on the day of challenge

**Figure 4:**
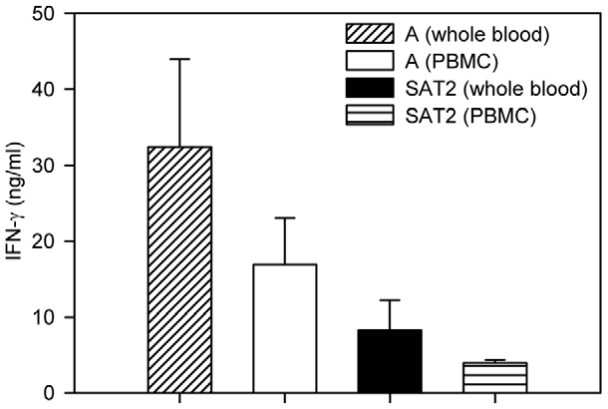
Comparison of mean IFN-γ responses in the re-stimulated whole blood and PBMC of cattle. Comparison of mean IFN-γ responses in the re-stimulated whole blood and PBMC of cattle immunised with full dose A Malaysia 97 and SAT2 Eritrea vaccines on the day of challenge. Error bars represent standard error of the mean. Although mean IFN-γ response was higher in the whole blood assay than the PBMC assay in both A May 97 and SAT 2 Eritrea experimental animals, they were not significantly different (P>0.05, 2-sample t-test) from each other.

In the full dose vaccinated groups of A Malaysia 97 and SAT2 Eritrea, although the IFN-γ response was always (post-vaccination and post-challenge) higher in the whole blood assay than in the PBMC induction assay (Fig. 4), the two results were not significantly different (P = 0.341 and P = 0.830). Addition of plasma prepared from 200 μl of heparinised blood of an animal to the PBMC cultured from the same amount of blood of that particular animal and induced over night with 2 μg of 146 s FMDV inactivated antigen resulted in the production of a similar amount of IFN-γ to the whole blood assay (data not shown).

#### 1.3.2 Comparison of IFN-γ production by whole blood re-stimulation assay with that of ELISpot assay

**Figure 5:**
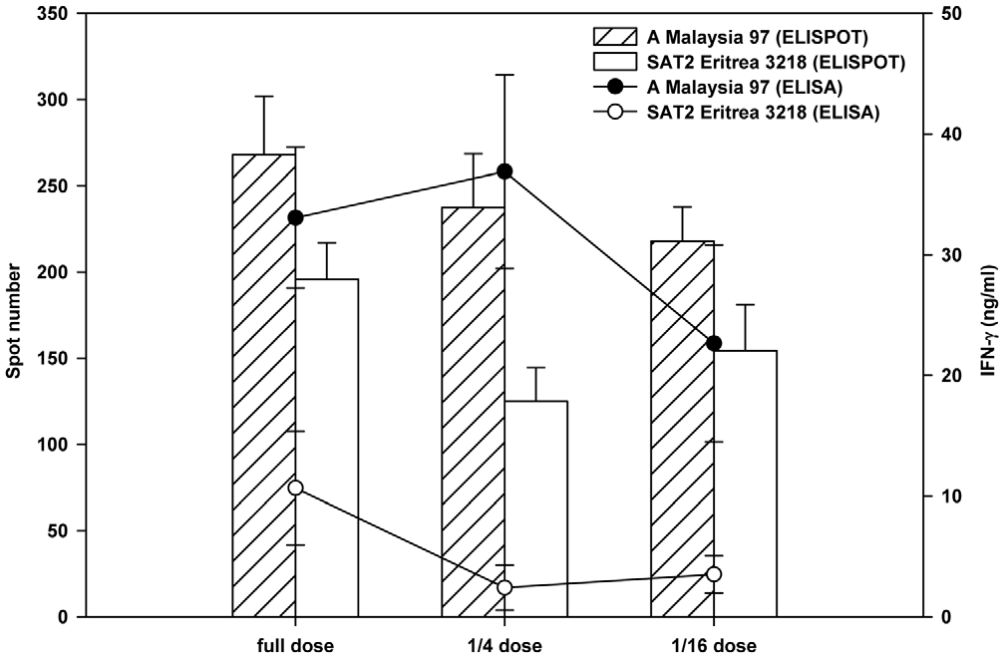
Comparison between the ELIspot assay and whole-blood induction assay. Comparison between the mean number of IFN-γ producing cells (bar) detected by ELIspot assay and the mean quantity of IFN-γ (line) detected by the re-stimulated whole blood assay in cattle immunised with A Malaysia 97 and SAT2 Eritrea vaccines on 19 days post challenge. Error bars represent standard error of the mean. The mean IFN-γ producing cells/quantity of IFN-γ production in ELIspot assay/re-stimulated whole blood assay were based on the mean data collected on 19 dpc. PBMC were seeded at 2×10^5^ per well. Error bars represent standard error of the mean.

For the ELISpot assay, PBMC were separated from all the animals in both experiments at 3 time points (0, 19 and 31 dpc) and re-stimulated with specific vaccine antigen *in vitro*. A significant difference (P<0.05) in IFN-γ producing cell spots was observed between the vaccinated and non vaccinated groups (ANOVA) in both the experiments similar to whole blood re-stimulation assay. The mean number of IFN-γ producing cells in ELISpot and mean quantity of IFN-γ produced in whole blood induction assay in the A Malaysia 97 and in the SAT2 Eritrea trial were compared (Fig. 5) by analysing 19 days post-challenge samples. A direct correlation (correlation coefficient = A Malaysia 97, 0.674 and SAT2 Eritrea, 0.576; A Malaysia, P<0.011and SAT2 Eritrea, P<0.025 estimated by Pearson's correlation coefficient) was found between the number of IFN-γ-producing cells in ELISpot and the quantity of IFN-γ produced in the supernatant of the induced whole blood (Fig. 5).

#### 1.3.3 Comparison of whole blood re-stimulation assay with Lymphocyte proliferation assay

**Figure 6:**
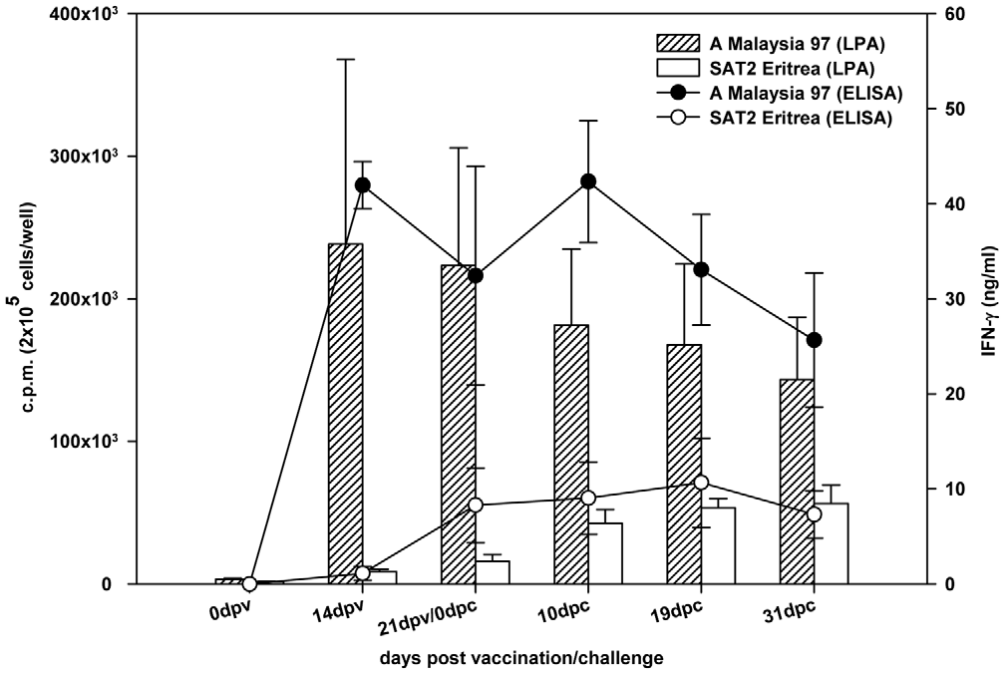
Comparison of Lymphocyte proliferative response and IFN-γ response with re-stimulation of specific vaccine antigen. Comparison of mean lymphocyte proliferation responses (bars) and mean IFN-γ responses in whole blood assay (lines) in A Malaysia 97 and SAT2 Eritrea full dose groups animals. Proliferation of re-stimulated PBMC measured by [^3^H] thymidine incorporation expressed as counts per minute (c.p.m.). PBMC were seeded at 2×10^5^ per well and incubated for 6 days. Error bars represent standard error of the mean.

A significant difference (P<0.05) in proliferative response between the vaccinated and non-vaccinated groups was only observed after 21 days post-vaccination (2-Sample T-test) in the case of the A Malaysia 97 experiment and at the 10th day post-challenge in case of the SAT2 Eritrea experiment; the vaccinated group showing a high proliferative response. The proliferative response in A Malaysia 97 vaccinated animals decreased gradually, even after the challenge in contrast to SAT2 vaccinated animals. The lymphocyte proliferative response was considerably lower in the SAT2 Eritrea potency test throughout the experiment compared to the A Malaysia 97 potency test. Interestingly, as in the whole blood IFN-γ assay, a significant difference (P<0.05) in the proliferative response was observed between the full dose groups of both experiments starting from the second week post-vaccination (2-sample t-test) apart from on the 19^th^ (P = 0.059) and 31^st^ (P = 0.072) dpc (Fig. 6). A good correlation (correlation coefficient = 0.368, P = 0.045) was observed between lymphocyte proliferation and IFN-γ production in whole blood assay in A Malaysia 97 experiments by Pearson's correlation coefficient. Similarly, a comparison between the proliferative responses and IFN-γ responses (whole blood assay) in full dose vaccinated animals of SAT2 Eritrea experiment showed a positive correlation between the assays (correlation coefficient = 0.531, P = 0.003).

### 1.4 Identification of the phenotype of the proliferating lymphocytes and IFN-γ producing T cells

#### 1.4.1 Determination of phenotypes of proliferating cells by carboxyfluorescein diacetate, succinimidyl ester (CFDA SE )staining and FACS analysis

**Figure 7:**
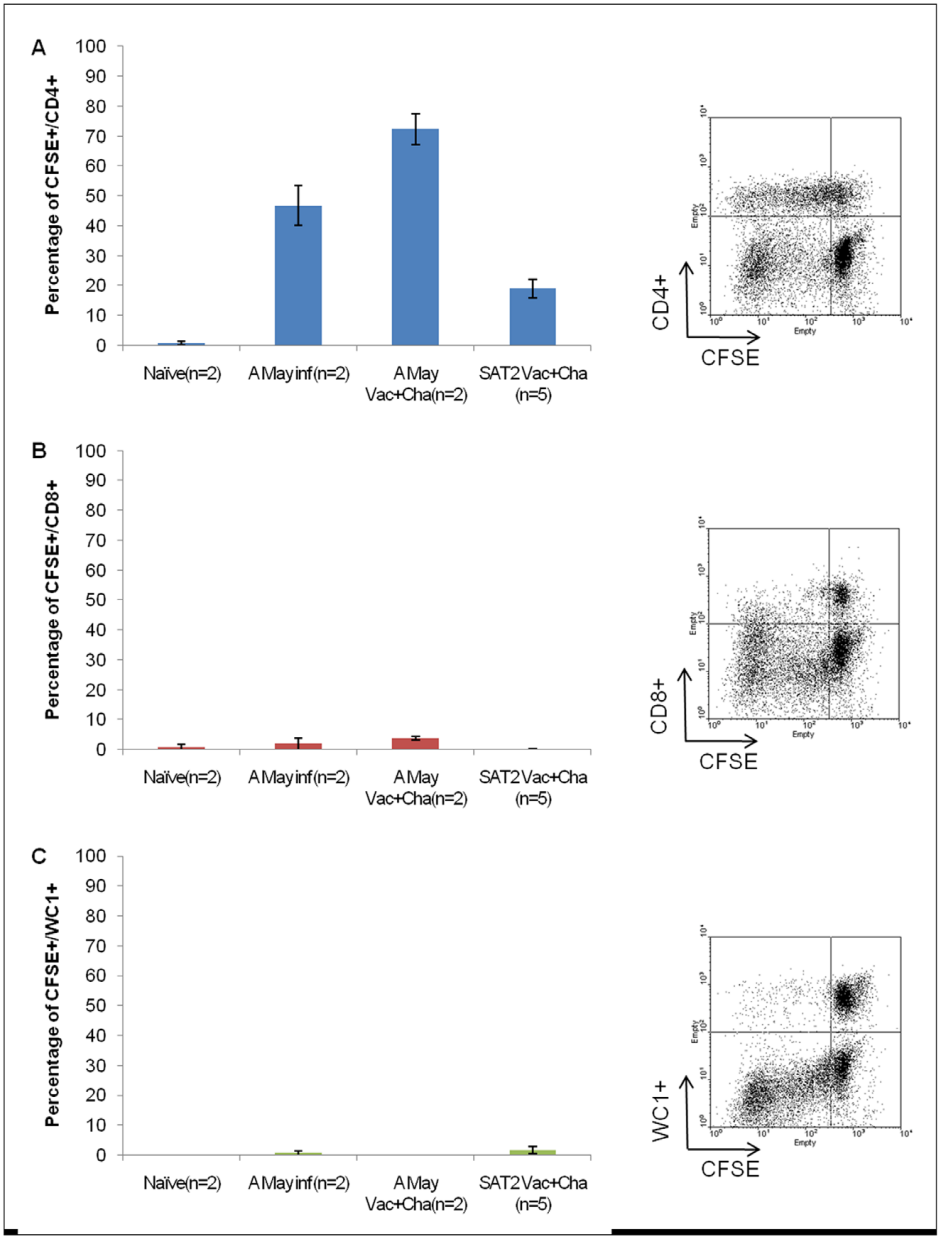
Determination of phenotypes of proliferating PBMC by FACS analysis. Determination of phenotypes (CD4+, CD8+ and WC1+) of proliferating PBMC by CFDA SE staining after re-stimulating with respective vaccine antigen and fluorescence-activated cell sorter (FACS) analysis. PBMC were separated from heparinised blood of vaccinated cattle originated from full dose vaccine group on 4 weeks after homologous FMDV challenge. Two naïve cattle without FMDV infection and two A Malaysia 97 FMDV infected cattle (4 weeks post-infection) without prior vaccination were included as control group. PBMCs from naïve animals were re-stimulated with SAT2 Eritrea vaccine antigen. FACS plots were exemplified only for one vaccinated challenged animal from SAT2 Eritrea animal experiment. Similar FACS plots were obtained for the PBMC originated from A Malaysia 97 and SAT2 Eritrea vaccinated animals. Error bars represent standard error of the mean.

The PBMC from two naïve animals stimulated with medium and vaccine antigen did not show any specific proliferative response for CD4+, CD8+ and WC1+ (Fig. 5A, B & C), whereas cells stimulated with mitogen showed high and similar proliferative responses for CD4+ and CD8+ and a low response for WC1+ cells (data not shown). Though similar responses were evident in the cells obtained from two non-vaccinated A Malaysia 97 FMDV infected animals on 28 dpc with positive mitogen re-stimulation (data not shown), the major proliferating population was found to be CD4+ cells with vaccine antigen re-stimulation (Fig. 7A, B & C). Similarly, in two A Malaysia 97 vaccinated and subsequently challenged animals (28 dpc), major proliferating cells were found to be CD4+ after re-stimulation with vaccine antigen (Fig. 7A, B & C).

**Figure 8:**
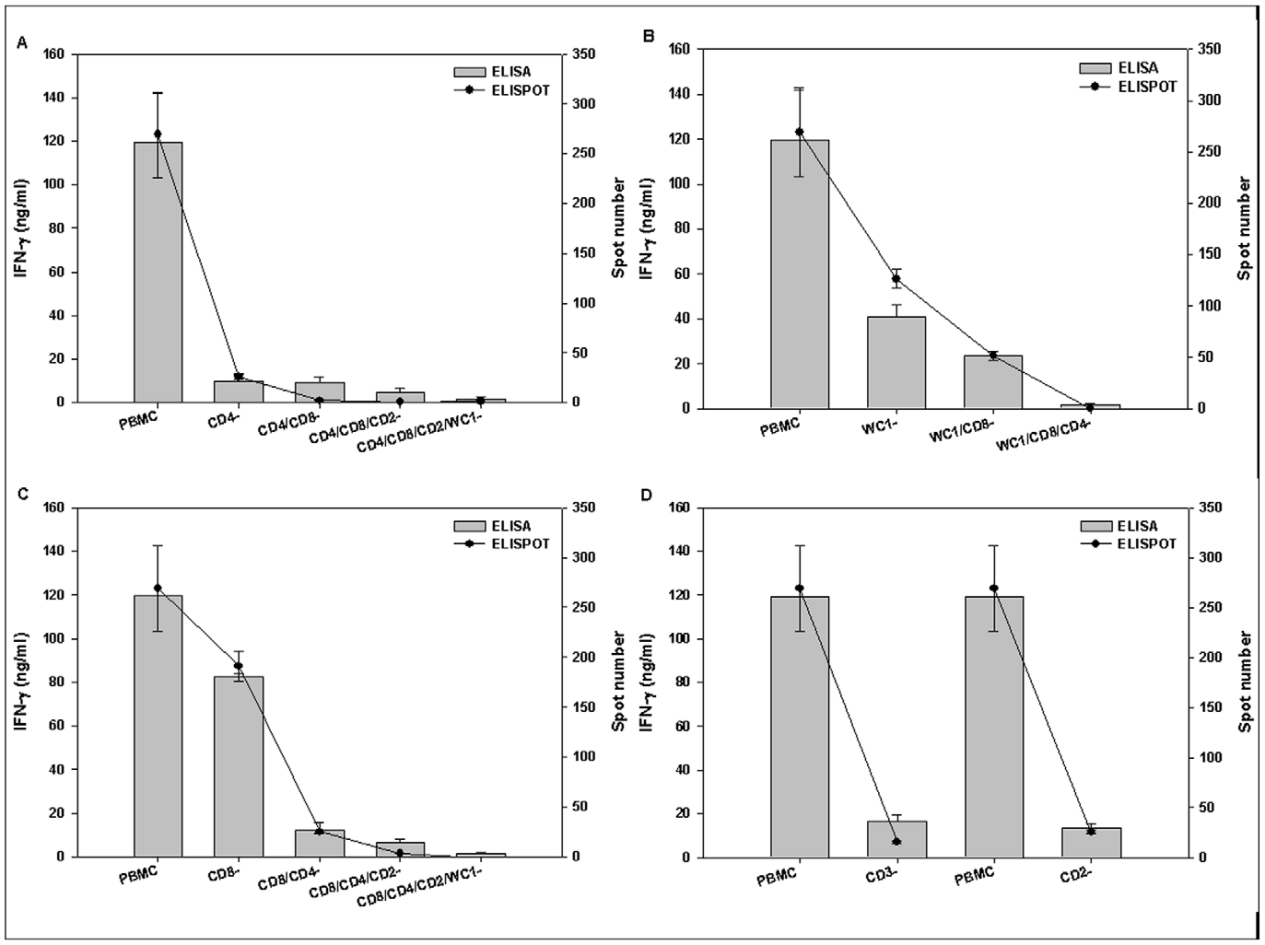
Measurement of IFN-γ responses from re-stimulated PBMC before and after depletion of various T-cell sub-sets. Measurement of IFN-γ responses from re-stimulated PBMC before and after depletion of various T-cell sub-sets by ELISA (bar) and ELISpot (line). PBMCs were separated from three A Malaysia 97 vaccinated and subsequently infected cattle on 29 days post-challenge. Each panel shows the quantity of IFN-γ levels before and after serial depletion of specific individual T cell population. A) whole PBMC → CD4 → CD8 → CD2 → WC1; B) whole PBMC → WC1 → CD8 → CD4; C) whole PBMC → CD8 → CD4 → CD2 → WC1; D) whole PBMC → CD3 and whole PBMC → CD2. Whole PBMC treated in the same way as depleted population. Error bars represent standard error of the mean.

CFDA SE and FACS analysis was carried out at various time points (0 dpv, 21 dpv/0 dpc, 16 dpc, and 28 dpc) for all five full dose SAT2 Eritrea vaccinated animals. During the vaccination period, no proliferation was observed upon mock and vaccine antigen re-stimulation (data not shown). Upon re-stimulation with mitogen, high proliferation of CD4+ and CD8+ cells and a moderate proliferation of WC1+ cells were observed (data not shown). Upon induction of vaccine antigen, proliferative response for CD4+ cells was significantly increased post-challenge in comparison to CD8+ and WC1+ cells (Fig. 7A, B & C) but not with the medium without antigen (data not shown).

A comparison of proliferating phenotypes of T cells in respect to vaccine antigen stimulation after 28 days post-challenge is depicted in figure 7. A significant difference of CD4+ proliferation (between CD4 & CD8; P = 0.005 and between CD4 & WC1; P = 0.006) was observed by the 2-t test in FMDV infected as well the FMDV vaccinated and subsequently infected animals. However, a significant difference of proliferation was not found between the CD8+ and WC1+ in FMDV infected as well FMDV vaccinated and subsequently infected animals (P = 0.683). A comparison of different proliferative phenotype responses among different groups of cattle (naïve, infected, Vaccinated and subsequently infected) was analysed by ANOVA which revealed a significant difference of CD4+ (P = 0.003) and CD8+ (P = 0.006) T-cell proliferations, where as no significant difference (P = 0.724) of WC1+ cells was observed. As in other assays, more CD4+ proliferation was observed in A Malaysia 97 vaccinated animals than the SAT 2 Eritrea vaccinated animals.

#### 1.4.2 In vitro depletion study

To identify which cells are responsible for IFN-γ production, various T-cell populations were depleted from whole PBMC using T-cell surface markers. Whole PBMC and PBMC depleted of a specific T-cell population using three rounds of negative sorts were induced with vaccine antigen and positive/negative controls, and IFN-γ was detected either by measuring IFN-γ production in cell culture supernatant, by ELISA, or by counting the number of IFN-γ producing cells by ELISpot.

The efficiency of the cell depletion was checked by FACS analysis. 10.9% of CD4+ cells were counted in the undepleted PBMC population whereas after depletion it was reduced to 0.6%. Similarly, CD8+ cells decreased from 15.3% to 1.9%, CD3+ cells decreased from 32.4% to 4.7% and WC1+ cells decreased from 32.2% to 0.5%, respectively. However, purity for CD2+ cells could not be assessed due to staining problems. Upon checking the purity by confocal microscopy, the specific stained cells were not seen in the depleted population though many of them were detected in un-depleted cell populations (data not shown).

The IFN-γ responses produced by PBMC before and after depletion of individual T-cell subsets were compared (Fig. 8). When CD4+ cells were depleted from the PBMC, the IFN-γ response was significantly decreased (P = 0.026, 2-sample t-test) compared with the amount measured in whole PBMC stimulation (Fig. 8A). A clear correlation was observed between the number of IFN-γ-producing cells (ELISpot) and the quantity of IFN-γ produced (ELISA) by Pearson's correlation coefficient (correlation coefficient = 0.997, P<0.001). Also, γδ T-cell (WC1+ cell) depletion led to a significant decrease of up to 1/3 in IFN-γ production (119.3 ng/ml to 40.9 ng/ml) in whole PBMC (P = 0.036, 2-sample t-test) (Fig. 8B) and a positive correlation was again observed between the two assays (correlation coefficient = 0.988, P = 0.012). On the other hand, depletion of CD8+ cells did not cause a significant reduction in the IFN-γ level (P = 0.067, 2-sample t-test) compared to CD4+ and WC1+ cell depletion (Fig. 8C). A direct correlation was also observed between the two assays (correlation coefficient = 0.999, P<0.001). Further depletion of CD4+ cells from the CD8+ depleted cell population caused a significant reduction in IFN-γ production (P = 0.047, 2-sample t-test) (Fig. 8C). As we did not have a direct surface marker (MAb to NKp46) for bovine NK cells, the difference in IFN-γ production between CD3+ (mature T-cell) and CD2+ (T-cell as well as NK cell) depleted population is thought to be caused by NK cells (Fig. 8D). No significant difference (P = 0.186, 2-sample t-test) in IFN-γ production could be established between CD3+ and CD2+ cell-depleted populations (Fig. 8D).

## Discussion

FMD is one of the most contagious diseases of livestock and causes severe economic losses in susceptible cloven-hoofed animals. Systematic vaccination of cattle led to a dramatic reduction in the number of disease outbreaks in Western Europe [16] and the successful eradication of the disease, led to the EU decision to abolish general vaccination of cattle against FMD and to adopt a non-vaccination policy in 1991 [16]. However, recent FMD outbreaks in a number of FMD-free countries have significantly increased public awareness of the disease [17] and the need for an emergency vaccination capability. More recently, we have seen an FMD outbreak in South Korea being controlled by vaccination with the aim of gaining FMD-free status.

The importance of the induction of virus neutralising antibodies to provide protection against FMDV infection is well known. However, specific antibody does not guarantee sterile immunity or clinical protection against FMDV [4]. Moreover, protection against FMDV infection has been observed in the absence of a detectable specific humoral response [18]. Consequently, it has been suggested that a cell-mediated immune response is also necessary for effective immunity to FMDV infection and for the control of the disease [19], [20]. Both CD4+ and CD8+ antiviral responses have been detected following FMDV infection, although the role of these responses in protection remains unclear [20], [21], [22], [23]. T helper cells have been reported to recognise a number of viral epitopes, located both in the viral capsid and viral non-structural proteins [1], [24]. Plasmacytoid dendritic cells (pDCs) interacting with immune complexed virus are the major source of type I interferon production during the acute FMDV infection [25]. It appears that T cell responses, mediated by CD4+ cells, are required for protective immunity against FMDV by participating in the production of antiviral antibodies [21], [23], [26], [27], [28]. IFN-γ expression is therefore frequently used to measure antigen-specific T-cell activation [29]. Van Lierop and colleagues [30] were the first to describe IFN-γ production from MHC II restricted T cells (CD4) in response to FMDV antigen and peptides. The peptides caused lympho-proliferation and were shown to induce a Th1 type response, irrespective of MHC haplotype involved. Gerner et al [8] also demonstrated the proliferation and IFN-γ production of lymphocytes from FMDV infected cattle when re-stimulated with a peptide representing the VP1 region of the structural protein of FMDV. A clear role of IFN-γ is to activate macrophages, resulting in increased phagocytosis, increased MHC class I and II expression, and the induction of IL-12, nitric oxide and superoxide production, all of which are important in the elimination of intracellular pathogens [31]. Subsets of CD4+ class II MHC-restricted T cells respond to activation by antigen-presenting cells and antigen by producing Th1 response (IFN-γ) and a Th2 (IL_4_, IL_5_ and IL_13_) response [5]. IFN-γ has antiviral activity against FMDV [11], [12], [13] and also promotes NK and macrophage activation that are likely to contribute to the control of FMDV replication and spread within the host [13]. Although CD8+ T-cell-mediated responses to FMDV have been reported [7], [26], [32], [33], their role in protection is not very clear [5].

The whole blood re-stimulation assay was developed for the diagnosis of tuberculosis as an alternative to the tuberculin skin test [34]. This assay measures IFN-γ production by ELISA after overnight exposure of heparinised whole blood to either *M. bovis* specific antigen or *M. avium* as a comparison. The whole blood IFN-γ assay is used as a measure of T-helper-1 activation of CD4+ and CD8+ T cells and can be readily used on a large number of samples [35]. We developed a whole blood re-stimulation assay for FMDV similar to tuberculosis and showed that this assay could be used to differentiate between vaccinated and vaccinated-and-infected animals [36]. Further, we reported the existence of a correlation between IFN-γ production and the establishment of the carrier state in cattle. These initial results prompted us to correlate the cell-mediated immune response and virus neutralising antibody titre with vaccine induced protection and secondly, to determine the phenotype of cells responsible for IFN-γ production.

In this study, blood samples were taken from two vaccine potency tests (A Malaysia 97 and SAT2 Eritrea) and analysed by the whole blood IFN-γ assay, ELISpot assay and lymphocyte proliferation assay after re-stimulation with inactivated vaccine antigen to measure the cell mediated immune response in FMD vaccinated and infected animals. A good correlation between the whole blood re-stimulation assay and the other above mentioned standard immunological methods are established. The IFN-γ response was significantly higher in A Malaysia 97 vaccinated animals than the animals vaccinated with SAT2 Eritrea. On the other hand, there was little difference in the neutralising antibody titres of the different groups from these two experiments. However, four out of 15 cattle were clinically affected in the SAT2 Eritrea potency test whereas all the animals in the A Malaysia 97 experiment were clinically protected. This was in spite of the fact that a higher antigen payload is used in the SAT2 serotype vaccine than in A serotype vaccine. Thermostability of vaccine antigen is crucial for the generation of immune responses in animals to achieve protection against FMDV [37], [38], [39]. The thermal stability of the 146S particles from the seven serotypes at 49°C differ considerably [40], while differences exist within serotype SAT2 at 37°C [41]. A contributory factor for better protection in A Malaysia vaccinated cattle may therefore be the relative instability of the SAT vaccine antigen *in vivo* compared with A serotype vaccine antigen [15]. It has also been reported that the SAT serotype vaccines, particularly the SAT2 serotype, are less immunogenic than serotypes O, A and C [41]. The CMI responses detected by many of the assays (whole blood IFN-γ assay and Lymphocyte proliferation assay) were higher for the A Malaysia 97 vaccinated animals compared to the SAT2 Eritrea animals and this might be a factor in the more consistent protection afforded by the A Malaysia 97 vaccine in all groups. Concentration of antigen has been shown to be a factor that may influence the differentiation processes of naïve CD4 T cells for the production of cytokines [42]. Therefore, though both A Malaysia 97 and SAT2 Eritrea antigens were delivered in a similar way, the lower stability of the SAT2 antigen might have result in it having less influence on the differentiation of CD4+ cells to produce IFN-γ responses. As a similar level of virus neutralising antibody were produced for both A Malaysia 97 and SAT2 Eritrea vaccinated animals, the other possible reason for the low level of IFN-γ response in SAT2 Eritrea vaccinated animals might be related to the virus strain dependent IFN-γ production.

Although the humoral antibody response is very important in conferring protection against FMDV infection [3], animals with high or medium neutralising antibody titre are not always clinically protected [4]. Our study supports this, since similar levels of VN antibodies were observed for both experiments, but four of the SAT2 animals were clinically affected and these animals showed little or no IFN-γ response. Furthermore, the clinically protected animals had higher IFN-γ levels and VN antibody titres than the clinically unprotected animals on the day of challenge. Thus, not only humoral antibody but also the cell-mediated immune responses do indeed seem to have a role in FMD vaccine induced protection. In our two vaccine challenge experiments, using only the VNT response on the day of challenge one could correlate protection with humoral antibody in 22 out of 30 cattle (20 protected animals with high VNT and 2 unprotected animals with low VNT). Similarly, considering only the IFN-γ response on the day of challenge one could correlate protection with IFN-γ responses in 23 cattle (19 protected animals with high IFN-γ and 4 unprotected animals with low or no IFN-γ response). However, combining both the tests, one could correlate either high VNT or high IFN-γ responses with protection for 24 animals, low VNT or low IFN-γ responses for 2 unprotected animals and low IFN-γ or high/medium VNT for another 2 unprotected animals. This leaves only two animals which were protected despite having both low IFN-γ and low VNT responses.

By measuring the mean value of IFN-γ production on the day of challenge in the A Malaysia 97 and SAT2 Eritrea vaccinated animals, a significant difference was observed between the clinically protected and infected cattle and similarly, between carrier and non-carrier animals. This positive correlation between carrier and non-carrier animals with IFN-γ production was shown earlier in an A Iran 96 potency experiment [36]. Further, a positive correlation between IFN-γ and VNT for vaccine induced protection was recorded on the day of challenge not only in the A Malaysia 97 and SAT2 Eritrea experiments, but also in many other vaccine-challenge experiments (data not shown); all the clinically-affected animals had significantly lower IFN-γ response than the clinically-protected animals. Further, we have recently shown that IFN-γ response from DNA vaccinated cattle correlated with protection and these protected animals also had high levels of neutralising antibody [43]. Therefore, we can suggest that the whole blood IFN-γ test may have potential in evaluating vaccine potency and may avoid the need for live virus challenge in potency tests [15]. Recently, Goris et al. [44], [45] highlighted the problem of high variability in the current European Pharmacopoeia vaccine potency test. Goris et al. [44] suggested that the variability can be reduced by increasing the number of animals per vaccine dose group to 25 instead of 5. However, this is highly impractical because of the need for a high biosecurity containment facility to house the animals and the high cost of the cattle. Moreover, animals must be challenged with virulent virus and so may suffer the clinical manifestations of the disease in the current potency test.

As in the whole blood IFN-γ assay, a significant difference (P<0.05) in the proliferative responses were observed between the full dose groups in both experiments from the first week post vaccination. It would have been very useful to have had an antigen-specific positive control such as ovalbumin (OVA) to ensure that the conditions of the cells and culture conditions were adequate to support antigen-specific proliferation of T cells. However, we could confirm that the IFN-γ response after re-stimulatiion with vaccine antigen was FMD-specific and not driven by a cellular component in both the vaccine and antigen used for restimulation, since there was no IFN-γ production when blood samples were induced with unrelated antigen (data not shown) and BHK cell culture lysate. To identify the type of proliferating lymphocytes, flow cytometric analysis was performed on samples from the A Malaysia 97 and SAT2 Eritrea potency tests. Using CFDA SE staining and FACS analysis, it was confirmed that it was mostly CD4+ T-cells that were proliferating after stimulation with FMD specific vaccine antigen. A smaller number of WC1+ T-cell (γδ T-cells) and CD8+ T cells were also seen to be proliferating, but only in vaccinated and subsequently challenged animals, confirming that the main IFN-γ producing cells are T-cells [46].

After depletion of the different subsets of lymphocytes, two *in vitro* assays (ELISA and ELISpot) were performed to compare the IFN-γ response between depleted and undepleted populations. A positive correlation was observed between the two assays which showed that the CD4+ T-cells are the main source of IFN-γ production when stimulated with FMDV antigen. However, depletion of γδ T-cells (WC1+ cell) from whole PBMC resulted in a considerable reduction of IFN-γ as they produce IFN-γ themselves and/or because they can act as antigen presenting cells (APCs) to the CD4+ T-cell population [12], [47]. CD8+ T-cells produced less IFN-γ in comparison to CD4+ T-cells and WC1+ T-cells, but, NK cells produced a negligible amount of IFN-γ. In tuberculosis and leprosy, similar amounts of IFN-γ were produced by CD4+ and CD8+-cells as detected by intracellular-staining using flow cytometry [48]. Vesosky et al. [49] showed that γδ T-cells, isolated from the peripheral blood of healthy cattle, could produce significant amounts of IFN-γ following stimulation with mycobacterial cell wall (from both *M. tuberculosis* and *M. bovis*). NK cells are known to be a major innate source of IFN-γ, which is produced rapidly during many infections before the development of an adaptive response [50]. Our study is consistent with the view of Szabo et al., [38] that although many cell types participate in combating infection, CD4+ T-cells critically determine the outcome of any given infection by producing IFN-γ.

It is clear from the present study that there is a positive correlation on the IFN-γ produced by CD4+ T-cells, and the virus neutralising antibody titre and the FMDV vaccine induced protection as well as the outcomes of FMDV persistence. This significant finding might help in the future design of new FMDV vaccines that may induce more CD4 Th1 response which we believe is essential for clinical protection as well as for the reduction of FMDV persistence in ruminants. TLR (Toll-like receptor) adjuvants tend to favour more Th1 CD4+ T-cell responses [51] and therefore selection of proper adjuvants for the new vaccines is vital.

## Materials and Methods

### Ethics Statements

The animal work was performed in a secure isolation unit at the Pirbright Laboratory, Institute for Animal Health (IAH). All the animal experiments were conducted in accordance with UK Home Office (HO) Rules, subject to approval by a local Ethics Committee. Procedures and end-points were defined in HO Project Licenses and were conducted by qualified persons as specified in their Personal HO Licenses.

### 1.1 Animal experiments and sample collection

#### 1.1.1 Animals

Holstein/Friesian cross-bred female cattle in good health, aged 6 to 9 months and averaging 200 kg in weight were used for these studies. They had never been vaccinated against or exposed to FMDV and antibodies against FMDV were not detected prior to vaccination or prior to challenge in the case of non-vaccinated control and donor animals.

All work was performed in a secure isolation unit at the Pirbright Laboratory, Institute for Animal Health (IAH).

#### 1.1.2 Vaccine challenge experiments

Two vaccine potency tests were carried out in compliance with the monograph of the European pharmacopoeia 04/2005: 0063′ Foot-and-mouth disease (ruminants) vaccine (inactivated)' [52]. These tests involved intramuscular vaccination with oil-adjuvanted inactivated FMDV vaccines; one test employed vaccine strains A Malaysia 97 and the other SAT2 Eritrea. The 17 cattle in each potency test were divided into four groups housed in separate boxes. Each of the first three groups contained five animals, whereas the fourth group contained only two animals, which served as unvaccinated controls. The first group of animals received a full dose (2 ml) and the second and third group received 1/4 (0.5 ml) and 1/16 (0.125 ml) of the full dose of vaccine, respectively. On the 21^st^ day after vaccination, all of the 15 vaccinated and the two control animals were challenged by virulent, cattle-adapted A Malaysia 97 or SAT2 Eritrea homologous virus [10,000 ID_50_/10^5^ TCID_50_ administered intradermally into two sites on the upper surface of the tongue (0.1 ml per site)]. The animals were then monitored over an 14-day period for any clinical signs of disease and maintained for a further 22 days thereafter. A potency of >6 PD_50_ was required for the vaccine to be accepted for emergency use.

#### 1.1.3 Sample collection

Heparinised and clotted blood samples (BD Vacutainer^TM^ LH 170 IU 10 ml or plain vacutainers, Becton Dickinson and Company, UK) were collected pre and post-vaccination and post-challenge. OP fluid samples were collected by probang cup and sub-aliquoted for storage at −80°C; either untreated for virus isolation or treated (200 μl OP fluid mixed with 300 μl of Lysis/Binding Buffer (Roche)) for detection of virus genome by real-time RT-PCR.

### 1.2 Detection of live virus/viral genome

#### 1.2.1 Cell culture and antigen detection

Aliquots of 200 μl of OP samples were inoculated onto monolayers of primary calf thyroid (BTY) cells for virus isolation [53].

#### 1.2.2 Quantitative real-time RT-PCR

Quantitative real-time RT-PCR (rtRT-PCR) was performed according to Reid et al. [54]. For the SAT2 Eritrea experimental probang samples, a new forward primer was used as described by Sammin et al. [55].

### 1.3 Antigen for re-stimulation assays

FMDV A Malaysia 97 and SAT2 Eritrea inactivated antigens used in the vaccine formulations, were also used in the re-stimulation assays excluding the adjuvant. These antigens were concentrated by ultra-filtration and then purified by industrial scale chromatography to remove extraneous proteins including the non-structural proteins (NSPs) of the virus [2]. The concentrated antigens were diluted 1/10 in sterilized PBS and centrifuged at 1000xg for 10 minutes at room temperature and then filtered through a 0.22 μm filter (Millipore, UK) to remove particulate matter.

### 1.4 Measurement of immune responses

#### 1.4.1 Virus neutralising antibody test (VNT)

Titres of neutralising antibodies against FMDV A Malaysia 97 and SAT2 Eritrea viruses were measured by micro-neutralisation assay as described in the OIE Manual of Diagnostic Tests and vaccines [53].

#### 1.4.2 Whole blood re-stimulation with specific antigen for determination of IFN-γ production

The quantity of IFN-γ production was measured after re-stimulation of whole blood with specific antigen [34], [36]. Briefly, for induction, 50 μl containing 2 μg of 146S FMDV inactivated vaccine antigen was added in duplicate to 200 μl of heparinised blood within four hours of collection in tissue culture grade 96-well plates. Negative control antigens, PPDA at 10 μg per ml or an equal volume of PBS were added in duplicate to 200 μl of blood. BHK cell lysates were prepared by a freeze-thawing method and used as a negative control. Poke weed mitogen (PWM) (6 μg/ml) was used to stimulate duplicate wells as a positive control for IFN-γ production. Finally, duplicates of 250 μl of whole blood were processed without induction to determine the quantity of IFN-γ present in unstimulated whole blood. The plates were incubated at 37°C for 24 hours in a humidified atmosphere within a 5% CO_2_ incubator. After incubation, 100 μl of the supernatant was harvested after centrifuging the plates at 290xg for 10 minutes at 4°C, and frozen at −80°C for future testing by ELISA.

#### 1.4.3 Peripheral blood mononuclear cell (PBMC) separation

PBMC were isolated from heparinised blood using Histopaque®-1077 (Sigma) density gradient centrifugation. Cells were washed twice with Ca/Mg-free PBS and re-suspended in complete medium (RPMI 1640, 5% foetal calf serum, 1% non-essential amino acids, 1 mM sodium pyruvate, gentamycin, β-mercaptoehthanol).

#### 1.4.4 IFN-γ ELISA

A bovine IFN-γ ELISA kit (BioSource Europe S.A., Belgium) was used to measure the quantity of IFN-γ in the harvested supernatant. The ELISA was carried out according to the manufacturer's instructions but using 25 μl of supernatant diluted with filtered deionised water to a final volume of 100 μl. IFN-γ standards containing differing, known amounts of IFN-γ were tested in duplicate in the first two columns of each ELISA plate and the quantity of IFN-γ in each unknown sample was estimated from the standard curve generated from these. For these standards, recombinant bovine IFN-γ protein (kindly provided by Dr Jayne Hope, IAH Compton Laboratory) was diluted in 100 μl of deionised water to a concentration of 6, 4.5, 3, 1.5, 0.9 and 0 ng/ml, respectively.

#### 1.4.5 IFN-γ ELISpot (enzyme-linked immunosorbent spot)

Blood samples were obtained from cattle involved in the two vaccine potency tests (strains A Malaysia 97 and SAT2 Eritrea) to determine the number of IFN-γ producing cells by ELISpot (enzyme-linked immunosorbent spot) analysis. 96-well MultiScreen plates (Millipore) were coated overnight at 4°C with 50 μl of mouse anti-bovine IFN-γ CC330 antibody (Compton Laboratory) at a concentration of 8 μg/ml in PBS. The plates were washed three times with PBS then 100 μl of separated PBMC (2×10^6^ cells/ml) were added in triplicate to each well with an equal volume of diluted 146 s inactivated vaccine antigen (2 μg/well) or PWM (0.5 μg/well) or without stimulant (media alone or BHK cell lysate) and incubated for 18 hours at 37°C in a 5% CO_2_ incubator. The next day, cells were lysed by adding distilled water for 10 minutes. The plates were washed three times with PBS and then 50 μl of biotinylated mouse anti-bovine IFN-γ CC302b antibody (Compton Laboratory) was added to each well at a concentration of 5 μg/ml in PBS and incubated for two hours at room temperature. The plates were washed three times with PBS. Then, 50 μl of streptavidin alkaline phosphatase conjugate (Caltag Laboratories) was added at a dilution of 1∶1000 in PBS and the plates were incubated at room temperature for another hour. After three washes with PBS, the presence of conjugated antibody was detected using alkaline phosphatase substrate (AP conjugate substrate kit, Bio-Rad). The reaction was stopped by washing the plates with distilled water. IFN-γ secreting cells were counted using the software program KS ELISpot (Release 4.8, Zeiss).

#### 1.4.6 Lymphocyte proliferation assay (LPA)

After separating the PBMC from heparinised blood, lymphocyte proliferation assays were carried out in 96-well round-bottomed micro-plates. This assay was performed on samples from animals that had received the full vaccine dose only and from non-vaccinated controls from the A Malaysia 97 and SAT2 Eritrea potency tests.

One hundred microlitres of PBMC (2×10^6^ cells/ml) in medium was added to each well. An additional 100 μl of medium or BHK cell lysate was added to the negative control wells. One hundred microlitres of diluted 146 s FMDV vaccine antigen (2 μg/well) or PWM (0.5 μg/well) were added as positive controls, each in triplicate wells. The PBMC were cultured for 5 days at 37°C in a 5% CO_2_ incubator and subsequently pulsed with 2 μCi/well of ^3^H-thymidine. After 18 hours incubation, cells were harvested on a Glass fibre filter (size 90×120 mm, Wallac) using a 96-well cell harvester (TOMTEC) and the radioactivity measured using a liquid scintillation counter (MicroBeta Wallac TriLux, Perkin Elmer).

#### 1.4.7 Carboxyfluorescein diacetate, succinimidyl ester (CFDA SE) staining and fluorescence-activated cell sorter (FACS) analysis

The percentage of individual T-cell phenotypes in the proliferating PBMC populations was determined by fluorescence-activated cell sorter (FACS) analysis. PBMC were separated from two animals given a full dose of A Malaysia 97 vaccine and challenged with homologous virus, both of which showed a good response in lymphocyte proliferation assays. PBMC were also analysed from two non-vaccinated but infected control animals from the same study, two FMDV naïve animals and from 5 animals vaccinated with a full dose of SAT2 Eritrea vaccine that were also challenged with homologous virus. After separating the PBMC, the cells were labelled with 5 μM carboxyfluorescein diacetate, succinimidyl ester (CFDA SE) in pre-warmed PBS containing the probe for 15 minutes at 37°C [56], [57], [58]. Cells were pelleted by centrifugation, re-suspended in fresh pre-warmed complete medium and incubated for another 30 minutes to ensure complete modification of the CFDA SE. One hundred microlitres of PBMC (2×10^6^cells/ml) were stimulated in triplicate wells in 96-well round-bottomed micro-plates with equal volumes of inactivated vaccine antigen (50 μg/well), PWM (5 μg/ml) or medium. After stimulating for 6 days, the cells were collected by pelleting at 250xg for 3 minutes followed by resuspension and double washing with FACS wash buffer (PBS containing 1% BSA, 0.1% sodium azide). Staining for different cell surface markers was carried out for 20 minutes on ice in the dark with the following antibodies, CC8 (Serotec) to identify CD4+ T cells [59], CC63 (Serotec) to identify CD8+ cells [60] and CC15 (IAH Compton Laboratory) to identify WC1+ γδ TCR+ cells [61]. PBMC were then washed twice with FACS wash buffer and incubated for 20 minutes on ice in the dark with a goat anti-mouse Ig-R-PE (phycoerythrin) conjugated secondary antibody (Southern Biotech). PBMC were finally washed twice in FACS wash buffer and analysed in a FACS calibur flowcytometer.

### 1.4.8 In vitro T-cell depletion assay

Various T-cell populations were depleted serially from the whole PBMC separated from three A Malaysia 97 vaccinated and subsequently infected cattle at 29 days post-challenge, and then induced with FMDV inactivated antigen to identify which cells were responsible for IFN-γ production. Briefly, separate aliquots of 1×10^7^ PBMC were pelleted by centrifugation at 250xg for 5 minutes at 4°C and were incubated with 250 μl (1 μg/ml) of a monoclonal antibody binding to specific cell surface markers for 10 minutes at room temperature. The following antibodies were used: CC8 (Serotec) to deplete CD4+ T-cells [59], CC63 (Serotec) to deplete CD8+ T-cells [60], CC42 (Serotec) to deplete CD2+ mature T-cells (CD4+, CD8+ and NK cells) [62], [63], MM1A (Bill Davis, WSU) to deplete CD3+ T-cells (CD4+ and CD8+ cells) [64] and CC15 (IAH Compton Laboratory) to deplete WC1+ γδ T-cells [61]. The cells were then washed twice by adding 20 ml of 1% BSA (bovine serum albumin, Sigma) in PBS followed by centrifugation at 250xg for 5 minutes at 4°C. The cells were then re-suspended in 2 ml of 1% BSA in PBS and coated with 100 μl of Dynabeads® Pan Mouse IgG (Invitrogen), which were previously washed three times with 1% BSA in PBS, for 30 minutes at 4°C with continuous rotation. Then specific T cell populations that bound to the beads were removed using a magnetic rack to deplete target cells. The depletion of each cell population was repeated three times to increase the efficiency of depletion of the target population. PBMC (2×10^6^ cells in 200 μl medium) before and after depletion of each cell population were induced with inactivated vaccine antigen (50 μl containing 2 μg of 146S) and negative/positive controls (PPDA 10 μg/ml, mock antigen, BHK cell lysate and PWM 6 μg/ml) for production of IFN-γ. Undepleted whole PBMC were treated in the same way as depleted PBMC except for the addition of monoclonal antibody binding to the specific cell surface markers.

#### 1.4.9 FACS analysis and confocal microscopy to check the purity of depleted T-cells

The purity of depleted cells was checked by FACS analysis. Briefly, PBMC and sorted cell populations were collected by pelleting, resuspending and washing in FACS wash buffer. Staining for different cell surface markers was performed for 20 minutes on ice in the dark with the same antibody used in depletion. Also, an aliquot of each cell population was incubated with second antibody alone. Cells were then washed twice with FACS wash buffer and incubated for 20 minutes on ice in the dark with goat anti-mouse Ig-R-PE-conjugated secondary antibody. Cells were finally washed twice in FACS wash buffer and analysed in a FACS calibur flow cytometer by counting 5000 gated cells.

### 1.5 Statistical analysis

Analysis of variance (ANOVA) was used to compare the level of IFN-γ production between the different vaccinated groups and the non-vaccinated control group in both vaccine potency experiments. When only two groups were involved, differences in mean values between groups were assessed through a pairwise comparison using the student t-test adjusted by the Bonferroni-Holm correction [65]. A difference was considered to be significant at P<0.05. Statistical analysis was performed using STATA 11.2 SE (StataCorp LP).
